# Prévalence et facteurs associés au tabagisme chez les adultes dans l’arrondissement de Moungali à Brazzaville en 2021

**DOI:** 10.11604/pamj.2022.43.6.34180

**Published:** 2022-09-05

**Authors:** Arkadit Jeandria Nkodia, Bermeland Ewuinh Tsiobinda, Jesse Saint Saba Antaon, Steven Richy Missongo, Prisca Doriane Ngnikam Tienkeu, Ceverly Hortège Dieudonné Milandou

**Affiliations:** 1 Association Congolaise pour la Santé publique et Communautaire, Brazzaville, République du Congo,; 2 Faculté de Médecine et des Sciences Biomédicales, Université Yaoundé I, Cameroun,; 3 Environnement Recherche Action, Yaoundé, Cameroun,; 4 District Sanitaire de Moungali, Brazzaville, République du Congo

**Keywords:** Tabagisme, adultes, prévalence, facteurs, Brazzaville, Smoking, adults, prevalence, factors, Brazzaville

## Abstract

**Introduction:**

l´épidémie du tabagisme pose un véritable problème de santé publique dans le monde et en Afrique. Notre étude avait pour objectif d´estimer la prévalence et d´identifier les facteurs associés au tabagisme chez les adultes vivant dans l´arrondissement de Moungali.

**Méthodes:**

nous avons réalisé une étude transversale à visée analytique. La taille de l´échantillon était de 800 adultes. Ils ont été sélectionnés par sondage aléatoire à deux degrés durant le mois de septembre 2021 dans l´arrondissement de Moungali, Brazzaville. La régression logistique binaire par la méthode pas à pas ascendante a permis d´identifier les facteurs associés. L´ajustement du modèle a été vérifié par le test de Hosmer Lomeshow. Les données ont été collectées par l´application Kobo Collect V1.30.1. Elles ont été analysées par le logiciel Stata 15.0.

**Résultats:**

la prévalence du tabagisme était de 4,63%, IC95% [3,37- 6,31]. Le genre masculin (ORa=8,36 IC95% [3,74-18,72], p-value < 0,0001), la consommation d´alcool (ORa=2,6 IC95% [1,11-6,11], p-value = 0,028), et l´exercice d´une activité professionnelle (secteur formel ou informel) (ORa=3,91 IC95% [1,16-13,11], p-value = 0,027) étaient les facteurs significativement associés au tabagisme.

**Conclusion:**

nous avons conclu que les hommes sont plus exposés à un risque lié au tabagisme actif par rapport aux femmes. Par ailleurs, la consommation de l´alcool, l´exercice d´une activité professionnelle dans le secteur formel ou informel étaient aussi les facteurs associés au tabagisme actif. Pour lutter efficacement contre le tabagisme chez les adultes, les actions de prévention doivent prendre en compte ces facteurs.

## Abstract (en)

**Introduction:**

tobacco epidemic is a real public health problem in the world and in Africa. The purpose of this study was to estimate the prevalence and identify factors associated with smoking among adults living in the Moungali district.

**Methods:**

we conducted a cross-sectional analytical study of 800 adults. Two-stage random sampling was used to select subjects in the Moungali district, Brazzaville, in September 2021. Binary logistic regression using the ascending stepwise method allowed identification of the associated factors. The adjustment of the model was verified by the Hosmer Lomeshow test. Data were collected via the Kobo collect V1.30.1 app. and analyzed with the Stata 15.0 software.

**Results:**

smoking prevalence was 4.63%, 95% CI [3.37-6.31]. Males (AOR=8.36 95% CI [3.74-18.72], p-value < 0.0001), alcohol consumption (AOR=2.6 95% CI [1.11-6.11], p-value = 0.028), and professional activity (formal or informal sector) (AOR=3.91 95% CI [1.16-13.11], p-value = 0.027) were factors significantly associated with smoking.

**Conclusion:**

this study highlights that smoking is a stronger risk factor in men than in women. In addition, alcohol consumption and professional activity in the formal or informal sector are also factors associated with active smoking. Preventive actions focusing on these factors are necessary to effectively fight smoking in adults.

## Introduction

La consommation du tabac pose un véritable problème de santé publique dans le monde. Les données les plus complètes sur les tendances mondiales du tabagisme mettent en évidence son énorme impact sur la santé mondiale [1]. En 2015, selon l´Organisation mondiale de la Santé (OMS) environ deux personnes sur dix de la population mondiale fument [2]. Le tabac est l´une des principales causes de mortalité évitable dans le monde [3]. Il est directement associé au décès de la moitié de ses consommateurs [4]. Chaque année, près de huit millions de décès sont directement liés au tabac. Plus de 7 millions de ces décès sont dus à l'usage actif du tabac, tandis qu'approximativement 1,2 million résultent de l´usage passif du tabac ou l'exposition de non-fumeurs à la fumée secondaire [5]. Près de 80% des plus d'un milliard de fumeurs dans le monde vivent dans des pays à revenu faible ou intermédiaire [6]. L´exposition au tabac conduit à de lourdes conséquences néfastes sur l´état de santé de ces consommateurs [7-9]. L´Afrique présente la plus grande menace en termes de croissance de l´épidémie du tabagisme [10]. Les statistiques prédisent que les tendances des cas de la tuberculose en Afrique augmenteront rapidement en raison du tabac [11]. En raison de sa croissance économique, démographique et de son climat législatif, l´Afrique est devenue un marché de choix pour l´industrie du tabac [12-16]. Le besoin des données descriptives et analytiques liées à l´usage du tabac figurent comme l´une des priorités pour la lutte anti-tabac dans les pays en voie de développement [17].

En République du Congo, une loi antitabac avait été adoptée en 2012. Elle interdit de fumer dans les lieux publics, la vente de produits du tabac aux mineurs, la publicité, le parrainage ou la promotion en faveur du tabac. Elle appelle aussi à opposer des mises en garde sanitaires sur les paquets de produits du tabac [18]. Une étude en population générale réalisée en 2006 à Brazzaville montrait une prévalence de 10,73% de la consommation du tabac chez les adolescentes avec une prédominance masculine [19]. Par ailleurs, il sied de constater une inexistence des données récentes sur la consommation du tabac chez les adultes en population générale. Ces conditions peuvent ainsi limiter l´élaboration des politiques publiques saines de lutte contre le tabagisme par le manque des données factuelles. Il est donc important de connaître les tendances épidémiologiques du tabagisme en République du Congo à partir des données récentes. C´est ainsi que notre étude se propose d´estimer la prévalence et d´identifier les facteurs associés à la consommation du tabac chez les adultes à Brazzaville: le cas des adultes vivant dans l´arrondissement de Moungali en 2021. Nos objectifs spécifiques sont: déterminer la prévalence du tabagisme chez les adultes vivant dans l´arrondissement de Moungali; identifier les facteurs associés au tabagisme.

## Méthodes

**Population:** la population d´étude était constituée des personnes adultes ayant vécu au moins une année dans l´arrondissement de Moungali. Pour chaque ménage, une seule personne était interrogée. Il s´agissait du chef de ménage ou de sa conjointe. Les adultes sélectionnés aléatoirement, ayant donné leur consentement à la participation à l´enquête ont été inclus. Les personnes susmentionnées se trouvant dans l´incapacité de répondre au questionnaire ont été exclues.

**Type, lieu et période d´étude:** il s´agissait d´une étude transversale à visée analytique réalisée dans l´arrondissement de Moungali pendant le mois de septembre 2021. Notant que la République du Congo compte 12 départements. Au sein de ces départements, on y trouve deux grandes métropoles dont l´une est la capitale économique et l´autre la capitale politique, respectivement Pointe-Noire et Brazzaville. La ville de Brazzaville est subdivisée en neuf arrondissements. L´arrondissement de Moungali fait partie des neufs que compte la ville. Cet arrondissement est subdivisé en neuf quartiers. Il couvre une superficie de 14,28 km^2^ et une population de 252.625 habitants en 2021 pour environ 58750 ménages et une densité de 16675,3 hab/km^2^.

**Echantillonnage:** un sondage aléatoire à deux degrés (échantillonnage à plusieurs degrés) a été réalisé. Les unités primaires étaient représentées par les zones de dénombrement (ZD) considérées comme des grappes. Au total, 40 grappes (ZD) réparties dans les différents quartiers (09) de Moungali ont fait l´objet de notre enquête. Dans chaque quartier, quatre ZD ont été tirés au hasard, soit six quartiers sur neuf (Anciens Combattants; CEG Matsoua; Marché de 10 frs; CEG de la paix; école de peinture de poto-poto; Plateaux des 15 ans). Les quartiers ayant le plus grand nombre des ménages et ZD, soit 3 quartiers sur 9, comptaient cinq à six ZD. Il s´est agi notamment de Moukondo (5 ZD); la poudrière (5ZD) et dix maisons (6ZD). A l´intérieur des grappes, la taille des ménages à collecter était de 20 ménages/ZD pour l´ensemble des 40 ZD tirées au sort.

**Taille de l´échantillon:** la formule de Daniel Schwartz a été utilisée pour déterminer le nombre nécessaire des participants à cette étude. Fixant ainsi l´effet de grappe à 2, le risque d´erreur à 0,05 (1,96) et le degré de confiance à 95%. Le P a été considéré à 50% dans le but d´avoir la plus grande taille d´échantillon possible et aussi en raison d´absence d´étude récente. Soit P=50% pour avoir un échantillon maximum.


$$n\geq Z^{2}\frac{p.q}{i^{2}}k$$

κ = taille minimale de notre échantillon; z= paramètre lié au risque d´erreur, fixé à 0.05 et correspondant à 1,96 dans la table des écarts réduits; i= marge d´erreur (i=0,05); P=prévalence du tabagisme; q=1-P, k=effet de grappe=2.

n= [(1,96)]^2^ 0,50.0,50/ [(0,05)]^2^ x 2 = 768 ; 768+4%(n) ; soit une taille de 800 ménages. Une marge estimative d´environ 4% a été ajoutée pour palier au taux de non-réponse.

**Collecte de données:** afin d´expliquer les relations entre les différentes caractéristiques sociodémographiques, environnementales, socioculturelles en lien avec le tabagisme, la théorie du comportement planifié a été utilisée [20]. Ce cadre conceptuel et théorique a permis d´identifier d´une part les variables à intégrer dans notre étude et d´autre part pour expliquer les facteurs associés au tabagisme. La collecte de données a été réalisée par une équipe de dix enquêteurs expérimentés et préalablement formés. La technique *LQAS* (*Lot Quality Assurance Sampling*) a été utilisée pour la sélection aléatoire des ménages et la couverture de la taille attendue pour chaque quartier et tout l´arrondissement [21,22]. Les données ont été saisies instantanément avec Kobo collect version 1.30.1. L´application était installée dans les tablettes des enquêteurs [23,24]. L´envoi des données au serveur central était instantané et réalisé au mois de septembre 2021.

**Variables:** les variables de cette étude ont été structurées en deux entités (variable dépendante et variables indépendantes). En ce qui concerne la variable dépendante, elle était la déclaration des participants au sujet du tabagisme actif (oui/non). Ce sont les adultes ayant déclaré comme étant fumeurs actuels au moment de notre enquête, quelle que soit la fréquence (quotidienne ou occasionnelle). Il s´agissait de savoir si l´individu fume de la cigarette ou pas. Cette approche a été abordée dans plusieurs travaux de recherche [25,26]. S´agissant des variables indépendantes, elles concernaient: répondant (chef de ménage/conjointe du chef de ménage); sexe du répondant (homme/femme); âge en année; statut matrimonial (célibataire; en couple/en union; marié(e); divorcé(e)/séparé(e)); niveau d´éducation (aucun, primaire, secondaire, supérieur); statut professionnel (chômeur, retraité, secteur formel, secteur informel); les caractéristiques socio-culturelles: pratique de la religion (oui/non); habitude de vie: consommation de l´alcool: (oui/non).

**Analyse des données:** les données collectées ont été analysées à l´aide du logiciel Stata 15.0. Le calcul des fréquences a été effectué pour les variables qualitatives. En ce qui concerne les variables quantitatives, le calcul de la moyenne et écart-type a été effectué. La prévalence de la consommation du tabac a été calculée et exprimée en pourcentage. En analyse univariée, chaque variable indépendante a été croisée avec la variable dépendante. Ceci, afin d´étudier les associations entre chaque variable indépendante et la variable dépendante. Les Odds ratio bruts avec leurs intervalles de confiance à 95% et les P-values ont été calculés. Le seuil de p a été de 0,05. En analyse multivariée, les variables significatives en analyse bivariée ont été intégrées dans le modèle de régression logistique [27-29]. La méthode pas à pas ascendante a été utilisée pour la régression logistique binaire [30]. Les Odds ratio ajustés avec leurs intervalles de confiance et p-value ont été calculés. Le seuil de significativité de p a été de 0,05. L´ajustement du modèle final a été vérifié par le test de Hosmer-Lemeshow [30-33].

**Considérations éthiques:** le protocole de recherche a fait l´objet d´une autorisation par les institutions sanitaires (N°105/MSP/CAB/DDSSaB/DSM/SEC). Les participants ont été au préalable informés de l´étude. Tous les enquêteurs ont reçu les autorisations avant la collecte des données dans les ménages. Le consentement éclairé de chaque participant a été obtenu avant l´administration des questions. Toutes les données ont été confidentielles et anonymes.

## Résultats

### Caractéristiques sociodémographiques des adultes enquêtés

Les caractéristiques sociodémographiques de notre échantillon sont décrites dans le Tableau 1. Notre étude a concerné un échantillon aléatoire de 800 adultes vivant dans l´arrondissement de Moungali. Les conjointes de chefs de ménage étaient les plus nombreuses à être interrogées, soit 52,12% (n=417) contre 47,88% (n=383) pour les chefs de ménage. Il y avait une prédominance des femmes, soit 67,75%, (n=542) par rapport aux hommes, soit 32,25% (n=258). L´âge moyen était de 43 ans. La plupart des ménages vivaient en couple ou union libre, soit 45,75% (n=366). Le niveau d´éducation prédominant était le niveau secondaire, soit 57,62% (n=461). Le plus grand nombre des ménages travaillaient dans le secteur informel, soit 57,75% (n=462). La consommation d´alcool était observée chez 57,88% (n=463) des adultes. La quasi-totalité des enquêtés pratiquait la religion, soit 96,50% (n=772).

**Tableau 1:** caractéristiques sociodémographiques des adultes enquêtés (n=800)

Variables	n	Pourcentage
Age (années), min; max: 18; 83		
Age médian (q1 ; q3) : 41 (32 ;52) ; Moyenne ± Ecart-type : 43±13,47		
**Répondant**		
Chef de ménage	383	47,88
Conjointe du chef de ménage	417	52,12
**Sexe**		
Homme	258	32,25
Femme	542	67,75
**Statut matrimonial**		
Célibataire	214	26,75
En couple, union libre	366	45,75
Marié	138	17,25
Séparé, Divorcé	30	3,75
Veuf	52	6,5
**Niveau d´éducation**		
Aucun	17	2,12
Primaire	55	6,88
Secondaire	461	57,62
Supérieur	267	33,38
**Statut Professionnel**		
Sans emploi	122	15,25
Retraité	54	6,75
Secteur formel	162	20,25
Secteur informel	462	57,75
**Religieux**		
Oui	772	96,5
Non	28	3,5
**Statut actif**		
Aucun/Retraité	176	22
Secteur formel/informel	624	78
**Alcool**		
Non	337	42,12
Oui	463	57,88

**Prévalence du tabagisme chez les adultes dans l´arrondissement de Moungali:** sur l´ensemble de 800 ménages interrogés, (n=37) avaient affirmé consommer le tabac. Soit une prévalence du tabagisme actif dans la population générale des adultes qui est de 4,63% IC95% [3,37 -6,31%]. Ces résultats sont décrits dans le Tableau 2.

**Tableau 2:** analyse univariée des facteurs associés, (N=800)

Variables	Tabagisme		OR brute	IC à 95%	p-value
	Oui n=37	Non n=763			
**Sexe**					
Femme	8	534	Référence		
Homme	29	229	8,45	[3,81-18,77]	< 0,0001
**Statut matrimonial**					
Célibataire	14	200	Référence		
En couple, union libre	18	348	0,74	[ 0,36 -1,52]	0,410
Marié	3	135	0,32	[ 0,09 - 1,13]	0,076
Séparé, Divorcé	2	28	1,02	[0,22 - 4,73]	0,979
Veuf	0	52	-	-	-
**Niveau d'éducation**					
Aucun	2	15	Référence		
Primaire	1	54	0,14	[ 0,01 - 1,64]	0,117
Sécondaire	22	439	0,38	[ 0,08 - 1,75]	0,212
Supérieur	12	255	0,35	[ 0,07 - 1,72]	0,198
**Statut Professionnel**					
Sans emploi	3	119	Référence		
Retraité	0	54	-	-	
Secteur formel	23	439	2,08	[0,61 -7,04]	0,240
Secteur informel	11	151	2,89	[0,79 -10,59]	0,109
**Réligieux**					
Oui	33	740	Référence		
Non	4	23	3,89	[1,28 -11,92]	0,017
**Statut actif**					
Aucun/Retraité	3	173	Référence		
Secteur formel/informel	34	590	3,32	[1,008 - 10,95]	0,048
**Alcool**					
Non	7	330	Référence		
Oui	30	433	3,27	[1,42 - 7,53]	0,005

### Facteurs associés au tabagisme chez les adultes de l´arrondissement de Moungali

#### Analyse univariée

En analyse univariée (Tableau 2), nous avons observé que les hommes avaient un risque significativement plus élevé d´exposition au tabagisme par rapport aux femmes, ORb=8,45 IC95% [3,81-18,77], p < 0,0001. Les non religieux avaient un risque plus élevé d´exposition au tabagisme par rapport aux personnes religieuses, ORb=3,89 IC95% [1,28-11,92], p=0,017. La consommation de l´alcool a été associée à un risque significatif d´exposition au tabagisme, ORb=3,27 IC95% [1,42-7,53], p=0,005. Les personnes en activité (secteur formel/informel) avaient aussi un risque plus élevé d´exposition au tabagisme par rapport aux personnes sans activité (Sans emploi/Retraité), ORb=3,32 IC95% [1,008-10,95], p=0,048. Les autres variables: le niveau d´éducation, la profession et le statut matrimonial n´avaient pas montré d´association significative.

#### Analyse multivariée

En analyse multivariée (Tableau 3), seules les variables qui avaient une p-value < 0,05 lors de l´analyse univariée étaient éligibles (sexe, religion, alcool, activité professionnelle). La régression ajustée a montré que les adultes de sexe masculin ou les hommes (ORa=8,36 IC95% [3,74-18,72], p-value < 0,0001), les adultes exerçant une activité professionnelle, secteur formel ou informel (ORa=3,91 IC95% [1,16-13,11], p-value = 0,027) et les adultes consommant de l´alcool (ORa=2,6 IC95% [1,11-6,11], p-value = 0,028), avaient un risque significatif de consommation du tabac. La statistique test de Hosmer Lomeshow (χ^2^ =2,56, p-value= 0,634) a montré un bon ajustement du modèle aux données.

**Tableau 3:** analyse multivariée des facteurs associés par la régression logistique binaire

Variables	OR Ajusté (Modèle 1)	IC à 95%	P-value	OR Ajusté (Modèle final)	IC à 95%	P-value
**Sexe**						
Femme	Référence			Référence		
Homme	8,13	[3,62-18,26]	< 0,0001	8,36	[3,74-18,72]	< 0,0001
**Religieux**						
Oui	Référence					
Non	3,24	[0,93 -11,29]	0,066			
**Statut actif**						
Aucun/Retraité	Référence			Référence		
Secteur formel/informel	4,59	[1,32 - 15,94]	0,017	3,91	[1,16 -13,11]	0,027
**Alcool**						
Non	Référence			Référence		
Oui	2,38	[1,004 - 5,65]	0,049	2,6	[1,11 - 6,11]	0,028

## Discussion

Notre étude a permis d´estimer la prévalence de la consommation du tabac chez les adultes vivant dans l´arrondissement de Moungali. De plus, d´identifier les facteurs associés au tabagisme. La prévalence du tabagisme est de 4,63%. Le genre masculin, la consommation d´alcool et l´exercice d´une activité professionnelle (secteur formel ou informel) sont significativement associés au tabagisme.

Dans notre étude, la moyenne d´âge des adultes enquêtés était de 43 ans (écart-type=13,47). Elle est légèrement supérieure à celle trouvée par Reda *et al*. en Ethiopie [34] qui était de 35 ans (écart-type=13,6) ou Colwell *et al*. en RDC soit 31 ans (écart-type=12,3) [35]. La prévalence du tabagisme était de 4,63% avec un intervalle de confiance à 95% allant de [3,37 à 6,31%] en population générale. Notre prévalence était inférieure à celle trouvée par Bonnechère *et al*. au Burkina Faso [36], Peltzer *et al*. en Afrique du Sud [37] et Giuliani *et al*. en Somalie [38]. Par contre, notre résultat se rapprochait à celui trouvé par Yawson *et al*. au Ghana [39], Desalu *et al*. au Nigeria [40] et Tafawa *et al*. en Afrique du Sud [41]. En analyse univariée, nous a trouvé que ceux qui n´avait pas de religion avait un risque plus élevé à la consommation du tabac. Autrement dit, le fait d´avoir une religion était un facteur protecteur contre la consommation du tabac. Des résultats similaires ont été trouvés dans plusieurs études [42-44]. Ces tendances peuvent s´expliquer par le fait que certaines religions n´adhèrent pas à la consommation du tabac. Ce qui donne un risque moins élevé du tabagisme aux religieux par rapport aux non religieux.

Dans notre modèle final, nous avons trouvé que le genre masculin était significativement associé à la consommation du tabac. Plusieurs études menées auprès des adultes ou des adolescentes ont trouvé des mêmes résultats: au Malawi [45]; au Kenya [46]; Indonésie [47], une revue dans quatre pays, en Malaisie, Roumanie, Argentine, Nigeria [48], en Égypte [49] et au Nigeria [50]. L´association du tabagisme avec l´activité professionnelle avaient été incriminé dans une étude menée en Éthiopie [51] et au Rwanda [52]. En ce qui concerne l´alcool, les résultats similaires ont été retrouvés en Tunisie [53]; au Burkina Faso [54] et en Éthiopie [55].

Les limites de notre étude concernent les biais qui pourraient être induits par les réponses des personnes enquêtées, notamment les biais d´information. La question du tabagisme étant sensible pour les femmes ou certains hommes. Nous pensons que certains adultes auraient pu donner des réponses les faisant paraitre beaucoup plus valorisantes dans la société. Ce biais de désirabilité sociale conduirait au biais de classement. C´est-à-dire, certains sujets interrogés pourraient dire non à la question du tabagisme et ainsi être classés dans le groupe des non-fumeurs alors qu´en réalité ils fument. Ce qui pourrait conduire à une sous-estimation de la prévalence du tabagisme dans cette population générale. Toutefois, la recherche avec les enquêteurs expérimentés en santé publique a permis de réduire au maximum ce biais.

## Conclusion

Notre étude a montré que la prévalence du tabagisme chez les adultes n´est pas négligeable. Nous avons aussi conclu que les facteurs de risque liés au tabagisme incluaient: le genre masculin, la consommation de l´alcool et l´exercice d´une activité professionnelle. Au regard de ces expositions pouvant être fatales sur le capital santé des adultes, il est donc nécessaire de renforcer la surveillance et la lutte du tabagisme dans tout le territoire national. Il s´agit notamment de veiller à la mise en application des mesures MPOWER adoptées par l´Organisation mondiale de la Santé. Par ailleurs, un accent continu sur la recherche scientifique doit être mis en place pour mieux documenter ce phénomène en République du Congo. Toutefois, les mesures de lutte et de prévention doivent être renforcées chez les personnes de sexe masculin, les consommateurs d´alcool et les personnes professionnellement actives.

### Etat des connaissances sur le sujet

- *Au niveau mondial, environ deux personnes sur dix fument;*
- *Le tabagisme est responsable de près de la moitié de décès de ses consommateurs;*
- *L´exposition au tabac conduit à des conséquences lourdes et néfastes sur l´état de santé*.

### Contribution de notre étude à la connaissance

- *La prévalence du tabagisme chez les adultes dans l´arrondissement de Moungali est de 4,63%;*
- *Le genre masculin est significativement associé au tabagisme;*
- *La consommation de l´alcool ainsi que l´exercice d´une activité que ça soit dans le secteur formel ou informel sont aussi significativement associés au tabagisme*.

## Remerciements

Les auteurs adressent leurs remerciements à tous les enquêteurs ayant participé à la collecte de données de cette étude. Il s´agit des étudiant(e)s de santé publique de Master 1 et Master 2 de la Faculté des Sciences de la Santé de l´Université Marien Ngouabi de Brazzaville (République du Congo) dont les noms suivent: Joséline Niyotwizeye, Yola Makosso-Mathos, Charel Gaï Ossengue, Biency Pea Engambe, Elcira Oba, Divine Emérence Mattondo, Bend Lema Missongo, Gautier Bikindou, Préfina Daslaine Matini et Dorveil Lapa Lapa.
